# Nursing profession in Africa: A study on work engagement and talent management practices in South Africa

**DOI:** 10.1002/puh2.87

**Published:** 2023-05-04

**Authors:** Mpho Lerotholi, Adéle Bezuidenhout

**Affiliations:** ^1^ Department of Human Resource Management University of South Africa Pretoria South Africa; ^2^ School of Organisations, Systems and People University of Portsmouth Portsmouth Hampshire UK

**Keywords:** Africa, healthcare, nurses, positive psychology, public hospitals, social exchange theory, South Africa, talent management, work engagement

## Abstract

**Background:**

The global shortage of nurses, intensified by nurses migrating to richer countries, creates a health crisis in developing countries. This study investigates how strategic talent management practices can improve the work engagement of professional nurses in public hospitals in South Africa.

**Methods:**

A cross‐sectional, self‐administered survey was conducted among professional nurses employed in three large public hospitals in the Gauteng Province of South Africa. We measured: (1) management commitment, (2) talent review, (3) workforce planning, (4) staffing, (5) talent acquisition, (6) talent development, (7) talent commitment and (8) talent retention. The sub‐dimensions of work engagement that were measured included: (1) vigour, (2) absorption and (3) dedication. Descriptive and inferential (multiple regression) analyses were performed. The gap analysis calculated the difference between the current and desired status means talent management item.

**Results:**

The gap analysis revealed the biggest discrepancies between the value nurses attach to and the current status of management commitment and the talent review and staffing processes. Multiple regression analyses confirmed that current status‐rating talent acquisition impacts dedication and absorption combined (*r* = 0.112, *p*‐value = 0.012 < 0.05) and management commitment impacts absorption and vigour combined (*r* = 0.26, *p*‐value = 0.026 < 0.05). The importance‐rating revealed talent development and commitment combined influences dedication and absorption combined (*r* = 0.092, *p*‐value = 0.038 < 0.05), and talent review and planning impacts absorption and vigour combined (*r* = 0.115, *p*‐value = 0.010 < 0.05). Age and job status had a significant effect on absorption–vigour.

**Conclusion:**

Hospital managers can improve the engagement of nurses by demonstrating their commitment to talent development, offering a diverse variety of development opportunities and ensuring a pipeline of new nurses by improving the attractiveness of the profession. The study shows how strategic talent management practices can serve to improve work engagement in the healthcare sector.

## INTRODUCTION

Complex work settings like healthcare [1], struggle to attract and retain sufficient professional nurses [2]. The severe global shortage of nurses [3] poses a risk to many countries’ post‐COVID recovery, including the United States of America, the United Kingdom [4] and Spain [5]. The scarcity was exacerbated by the COVID‐19 pandemic [4, 5, 6, 7, 8, 9]. In response, wealthy countries recruit migrant nurses from Africa and Asia, contributing to the scarcity in developing countries [8]. Little is known about talent management in the nursing profession [9], and how it could be used to address the staffing crises [10]. Strategic talent management can improve nurses’ engagement and curtail high turnover during the post‐COVID recovery period [11].

### Talent management

A common approach to effective talent management does not exist [12]. Four talent management streams have been developed thus far [13]. The first views talent management as synonymous with regular human resource management procedures [14]. The second emphasizes talent pools, succession planning and the filling of high‐level positions [15]. The third stream has a traditional view of talent management, with two distinct emerging perspectives‐inclusive and exclusive. The ‘inclusive approach’ considers all employees valuable, and deserving of development opportunities [16], whereas the ‘exclusive approach’ focuses on selected high‐potential performers [17]. The fourth stream identifies talent management's contribution to organizational competitive advantage [18].

Talent management strategies are context‐specific, more complicated and less well understood in the resource‐scarce public healthcare service [1, 15]. In one context, it may imply long‐term sustainability, whereas in another, it may mean developing high‐potential people. It is noted that in some settings there is rivalry for talented nurses, increasing the need to create an engaging work environment [8]. Key talent management processes include management commitment, talent review, workforce planning, staffing, talent acquisition, talent development, performance management and talent retention [19].

### Work engagement

Engagement is defined differently by academics and practitioners, leading to some confusion [20]. Personal engagement refers to using one's physical, cognitive and emotional capabilities to perform one's jobs well [20]. Although ‘employee engagement’ and ‘work engagement’ are frequently used interchangeably, work engagement relates to employees’ feelings about the job (e.g. nursing), whereas employee engagement refers to feelings about the organization (e.g. the hospital). The most widely accepted definition describes work engagement as a positive, fulfilling, work‐related state of mind characterised by vigour, dedication and absorption. [7] Vigour refers to high levels of energy, resilience and determination [21]. Dedication refers to devoting effort and time [22] and absorption to focus and involvement in one's tasks [23].

This study uses ‘work engagement’ as something referring to the nurses’ perceptions about their important, but demanding jobs. Job demands and resources are major drivers of work engagement [24, 25]. As nurses have intense contact with patients in resource‐scarce hospitals [26] they offer an appropriate and interesting research context. Hospital managers need to understand how to encourage nurses’ work engagement by identifying context‐specific engagement drivers.

Social exchange theory describes how mutual obligations between parties in a reciprocal interdependent relationship are governed by ‘rules of engagement’ [27, 28]. When management is committed to talent management, relationships, engagement and organisational outcomes improve and turnover decrease [29]. Positive psychology emphasizes the importance of making peoples’ lives more fulfilling, [30] by nurturing their talent [31] and valuing growth and potential [32]. In Indonesia, research supported the link among talent management, work engagement, satisfaction and improved performance in nurses [33]. In Canada, the link among nurses’ resources, staffing and a manageable workload has been confirmed [34]. Talent management practices, therefore, hold promise to attract and retain people in the nursing profession [35].

This research aimed to conduct a Status‐Importance gap analysis of different strategic talent management practices, their importance and their effect on the work engagement of professional nurses in public hospitals in South Africa. The study also aimed to identify the effect of these talent management practices and nurses’ demographic variables on their work engagement.

**FIGURE 1:**
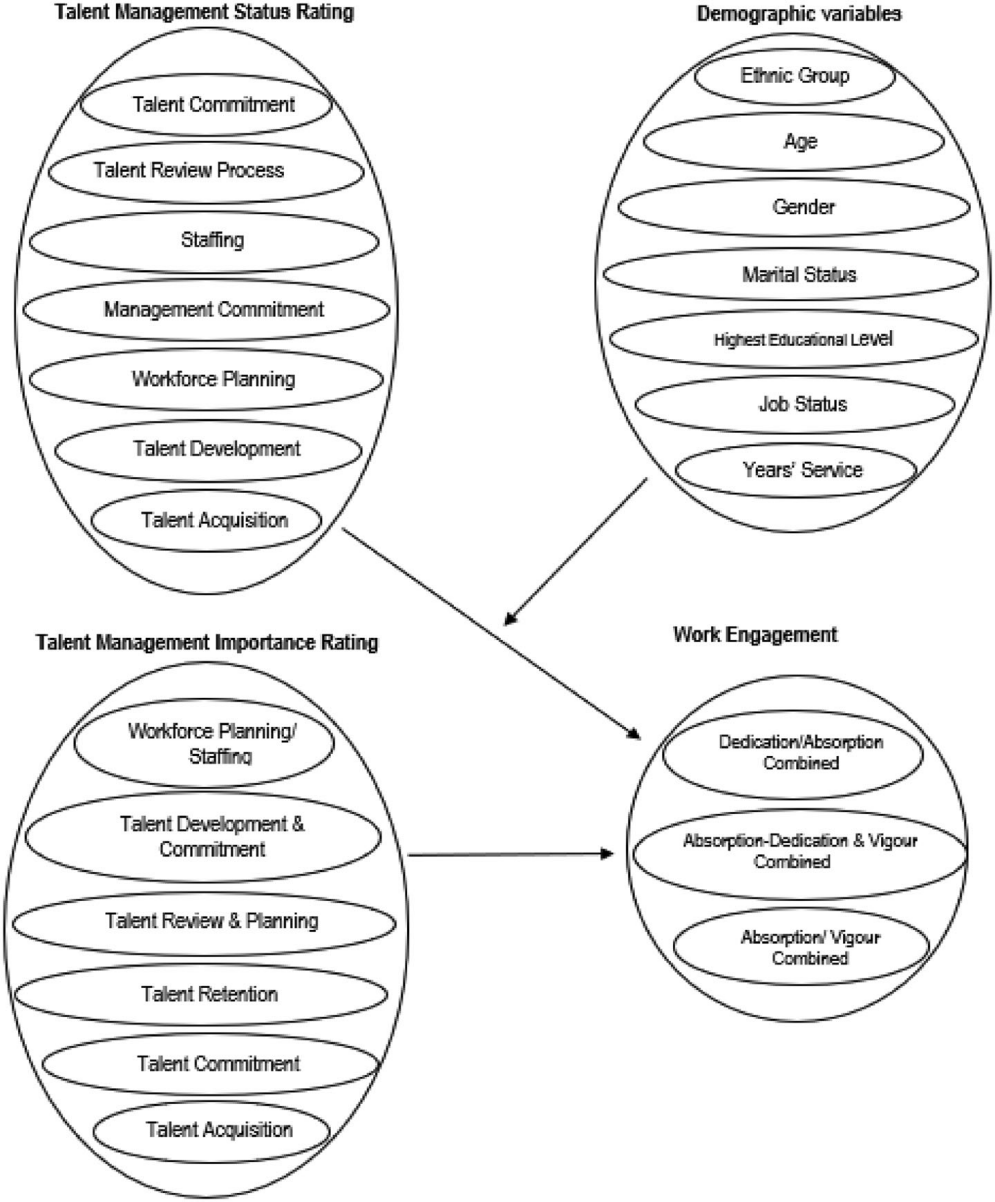
A conceptual framework of the proposed model.

Based on the discussion, a conceptual framework is presented (Figure 1). The framework shows that strategic talent management practices (status rating) have a positive effect on nurses’ work engagement. Strategic talent management practices (importance rating) have a positive effect on nurses’ work engagement. Demographic characteristics influence nurses’ work engagement.

## METHODS

### Research design, study population and setting

A cross‐sectional, self‐administered survey design was used to conduct the study on professional nurses employed in three large public hospitals situated in the Johannesburg and Pretoria region in the Gauteng Province of South Africa. The three academic hospitals included Steve Biko and Weskoppies Academic Hospitals situated in Pretoria, the capital city of South Africa and Helen Joseph Academic Hospital in Johannesburg. The population considered consisted of approximately 2800 professional nurses employed by these hospitals. Assistance and permission from the human resource departments were sought. The inclusion and exclusion criteria were as follows: Professional nurses on full‐time permanent contracts were included, whereas nurses employed by external agencies or on temporary contracts were excluded from the study. We distributed 700 questionnaires in this study.

### Study variables and instrument

Existing standardized questionnaires were adapted and combined to form the overall instrument. The instrument consisted of (1) sociodemographic variables questionnaire, (2) Talent Management Index and (3) the Utrecht Work Engagement Scale‐17 (UWES‐17) [2, 36]. The Talent Management Index consists of 35 items measuring eight different talent management practices [37] validated for the South African context [38]: (1) management commitment (five items), for example *The management of the hospital has a deep belief that talent is the key to competitive success*; (2) talent review process (five items), for example *Rewards and opportunities are provided to talented employees, based on their contribution*; (3) workforce planning (six items), for example t*alent supply and demand of employees over the next 2 years in our section have been forecasted*; (4) staffing (three items), for example *We are staffed at the rights levels in our section*; (5) talent acquisition (four items), for example *The hospital employment brand is strong and compelling among prospective employees*; (6) talent development (four items), for example *Coaching, mentoring and challenging assignments are primary development approaches in the hospital*; (7) talent commitment (four items), for example *The majorities of employees in my department/section are fully committed to the hospital* and (8) talent retention (four items*)*, for example *The reason people leave, especially top performers, are recorded and addressed*.

Respondents were first required to indicate the extent of their agreement on a five‐point Likert scale ranging from strongly disagree (1) to strongly agree (5) regarding the current status of the talent management practices and second, to indicate the importance of the talent management practices from not important (1) to crucially important (5).

Work engagement was measured using the UWES‐17 [2, 36], with three different sub‐factors, including (1) absorption (six items): for example *When I am working*; (2) dedication (five items): for example *I find the work that I do full of meaning and purpose* and (3) Vigour (six items): for example *at my work, I feel bursting with energy* [2]. The reliability of the instruments in a South African context has been established in previous studies [39].

### Data analysis

Descriptive analysis, including a Status‐Importance gap analysis and Cronbach alpha scores, factor analysis (principal component analysis with varimax rotation) and confirmatory factor analysis were calculated. Loadings were between 0.626 and 0.935 on the respective factors. The average factor loading was 0.666, indicating significant and nontrivial factor loadings. We controlled for demographic variables, specifically age, as it has been shown to influence work engagement [39]. Consequently, multiple regression analysis was conducted to examine whether any of the talent management practices do indeed predict work engagement. The confidence interval level was set at 95% (*p* ≤ 0.05) and the practical effect size at *r* ≥ 0.30 ≥ 0.50 (medium to large effect). Hierarchical regression analyses were then performed to calculate the relationships as presented by the framework. We investigated the relationships in more depth, as follows: Model 1 presents the effect of talent management status‐rating on dedication and absorption combined; Model 2 is about the effect of talent management status‐rating on absorption–dedication and vigour combined; and Model 3 is about the effect of talent management status‐rating on absorption and vigour combined. We also examined the effect of demographic variables on dedication and absorption combined in Model 4; demographic variables on absorption–dedication and vigour combined in Model 5; and finally the effect of demographic variables on absorption and vigour combined in Model 6.

### Ethical considerations

The researchers obtained ethical clearance from both the ethics committees of Unisa and the University of Pretoria, respectively, as well as permission from each of the three targeted hospitals. The project adhered strictly to ethical protocols such as obtaining informed consent and protection of anonymity. In terms of expectations of data sharing the data that informed the findings of this study are available in the supplementary material submitted to support this article.

## RESULTS

### Profile of respondents

Out of 700 questionnaires distributed, 504 were returned (response rate = 72%). The participants comprised predominantly young black (65%) females (73%) between the ages of 22–29 (53%) and 30–39 (23.9%). The respondents were permanently appointed (98, 4%) and most had more than 10‐year experience (64%). Overall, 14% had between 3‐ and 10‐year of work experience.

The results indicated modestly high scores for the talent management practices‐status rating (TMSR) (mean min = 3816, mean max = 4.09) talent management practices—importance rating (TMIR) (mean min = 4.05, mean max = 4.19) and work engagement.

**FIGURE 2:**
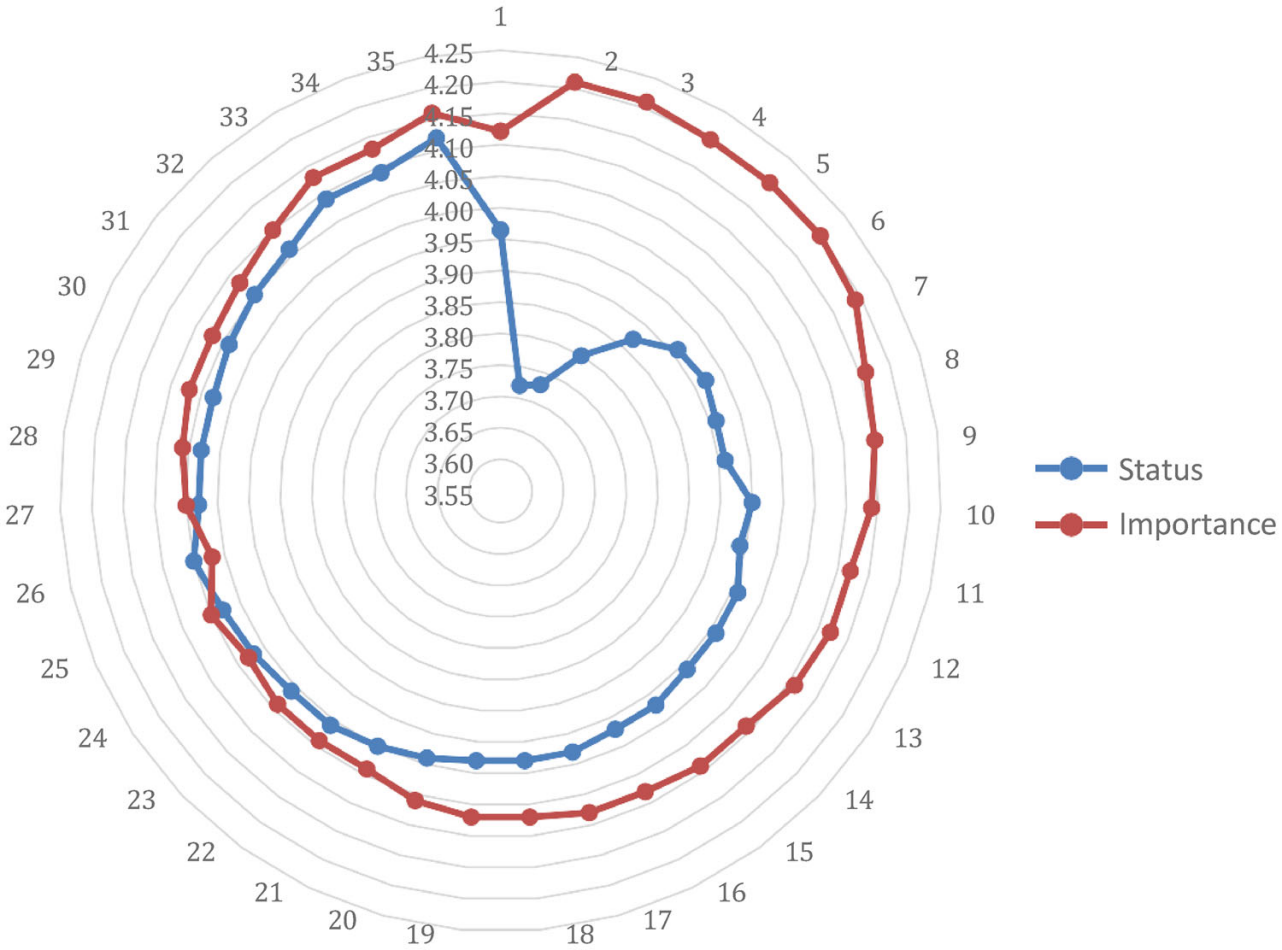
Importance‐Status gap analysis of strategic talent management practices.

A Status‐Importance gap analysis was performed per item and a visual presentation of the discrepancies between the nurses’ perception of the current status TMSR versus the importance of the talent management practices TMIR is presented in Figure 2. Gaps were calculated for each item.

The biggest gaps or discrepancies are visible from items 1 to 5. This corresponds with the first theoretical factor covered in the questionnaire, management commitment (items 1–5) and the second theoretical factor, the talent review process (items 6–10). The gap noticeably starts to decrease from workforce planning (items 11–16), and staffing (items 17–19), and eventually, talent acquisition (items 20–23) shows the two lines converging, implying that the current status of talent acquisition meets the respondents’ perception of the importance of this practice. This trend continues throughout the last three factors, talent commitment, development and retention. A closer inspection of the specific item discrepancies revealed the most serious deficiencies to be related to whether the hospital management is actively engaged with talent management practices, setting high standards and reinforcing positive results.

Abbreviations: TMIR, talent management practices‐importance rating; TMSR, talent management practices‐ status rating.

The results of the discrepancy gap analysis presented in Table 1 indicated moderate scores for the TMSR (mean = 3.47) and high scores for TMIR (mean = 4.102), indicating that nurses perceive more should be done to improve talent management practices in public service hospitals. Cronbach's alpha coefficients of all the components of talent management indicated good composite reliability, with scores between 0.81 and 0.95 [40]. To improve the model fit, the Cronbach alpha coefficient of WE needed consideration, and items that did not meet the criteria were deleted. Three sub‐dimensions dedication–absorption combined, absorption–dedication and vigour combined and absorption–vigour combined were retained. Principle component factor analysis found that eight factors of TMSR explained 76.41%; six factors of the TMIR rating explained 77.66%; and work engagement explained 49.76% of the variance, respectively. Factors that did not meet the required reliability were excluded from further analysis.

**TABLE 1:** Discrepancy gap analysis talent management status‐rating versus importance‐rating.

Original theoretical talent management practices	TMSR	TMIR	GAP
Management commitment	3.816	4.188	**−0.372**
Talent review process	3.92	4.168	**−0.248**
Workforce planning	3.96	4.102	−0.142
Staffing	4	4.073	−0.073
Talent acquisition	4.0025	4.045	−0.043
Talent commitment	4.0325	4.0375	−0.005
Talent development	4.0375	4.07	−0.033
Talent retention	4.09	4.13	−0.041

*Note*: The bold numbers show the biggest gaps.

The factor loadings of the majority of the constructs are above 0.5 [41], and the average variance explained (AVE) of all constructs is above 0.5, indicating convergent validity in all the items retained in the final measurement model. The discriminant validity of the measurement model, assessed through a comparison between the square root of the AVE and the highest inter‐construct correlation, indicated no concerns [42].

The constructs and the relationships illustrated in the framework fit the data satisfactorily, as the square root of the AVE values is above the inter‐construct correlations [12, 42]. Correlation and regression analysis results between TMSR and the WE sub‐dimensions, and the TMIR and WE sub‐dimensions were calculated. The TMSR results showed talent acquisition current status‐rating impacts dedication and absorption combined (*r* = 0.112, *p*‐value = 0.012 < 0.05) and management commitment status‐rating impacts absorption and vigour combined (*r* = 0.26, *p*‐value = 0.026 < 0.05).

For the TMIR, talent development and commitment combined influences dedication and absorption combined (*r* = 0.092, *p*‐value = 0.038 < 0.05). Talent review and planning impacts absorption and vigour combined (*r* = 0.115, *p*‐value = 0.010 < 0.05). This means that talent development and talent commitment (combined), as well as talent review and planning, are very important to support nurses’ engagement.

A summary of the regression analysis results for TMSR in Table 2 shows Model 1 (dedication and absorption) is statistically significant at 1% and 5% levels of significance (*R* = 0.045 with a *p*‐value of 0.000, *p*‐value <0.01) indicating that 4.5% of the total variation in dedication and absorption combined is explained by TMSR factors.

**TABLE 2:** Regression summary: talent management practices‐status rating (TMSR) and the three work engagement sub‐factors.

Dependent Variable	ANOVA^a^	*R* ^2^	Adjusted *R* ^2^	*F*	Sig.
De and Abs^a^	Model 1	0.059	0.045	4.418	0.000^d^
Abs, De and Vi combined	Model 2	0.010	0.004	0.731	0.646^d^
Abs and Vi combined ce	Model 3	0.024	0.011	1.772	0.091^d^

^a^ Dependent variable: WE—dedication and absorption combined.

^b^ Dependent variable: WE—absorption, dedication and vigour combined.

^c^ Dependent variable: WE—absorption and vigour combined.

^d^ Predictors: (Constant), talent commitment, talent review process, staffing, management commitment, workforce planning, talent development and talent acquisition.

Model 2 (absorbtion–dedication and vigour combined), as well as Model 3 (absorption and vigour combined), are not statistically significant at a 5% level of significance, explaining less than 2,5% of the total variation in WE and were not included in further analysis. Because the results of Model 1 (dedication and absorption combined) were significant, it was analysed further (Table 3).

**TABLE 3:** Regression summary for Model 1: talent management practices‐status rating (TMSR) and dedication‐and‐absorption combined.

	Unstandardized coefficients		Standardized coefficients		
Model 1	*B*	Std. error	Beta	*T*	Sig.
(Constant/independent variables)	3.040	0.354		8.582	0.000
Talent commitment	0.055	0.064	0.043	0.859	0.391
Talent review process	0.132	0.071	0.083	1.866	**0.063**
Staffing	−0.075	0.053	−0.068	−1.418	0.157
Management commitment	−0.033	0.027	−0.057	−1.222	0.222
Workforce planning	0.178	0.061	0.136	2.932	**0.004**
Talent development	−0.044	0.037	−0.057	−1.190	0.235
Talent acquisition	0.209	0.077	0.129	2.718	**0.007**

*Note*: The bold numbers show the most significant effects.

Model 1 contains the seven TMSR parameters (talent commitment, talent review process, staffing, management commitment, workforce planning, talent development and talent acquisition) and indicates the effect of these on the dedication‐and‐absorption combined dimension. The results show the talent review process, workforce planning and talent acquisition have a significant positive contribution to the model.

Talent management practices have a positive and significant effect on work engagement, as its *p*‐value (0.003) confirms the statistically significant effect of nurses’ perception of current talent management practices on work engagement.

The effect of demographic characteristics on the work engagement sub‐factors was investigated as presented in Table 4. Models 4 to 6 contained seven parameters, namely years of service, race group, gender, job status, highest educational level, marital status and age group.

**TABLE 4:** Regression summary demographic variables on work engagement factors.

Dependent Variables	ANOVA^a^	*R* ^2^	Adjusted *R* ^2^	*F*	Sig.
Model 4 De and Abs comb^a^	Regression	0.011	0.003	0.788	0.598^d^
Model 5 Abs‐De and Vi comb^b^	Regression	0.029	0.015	2.077	0.044^d^
Model 6 Ab and Vi comb^c^	Regression	0.044	0.031	3.2582	0.002^d^

^a^ Dependent variable: WE—dedication and absorption combined.

^b^ Dependent variable: WE—absorption—dedication and vigour combined.

^c^ Dependent variable: WE—absorption and vigour combined.

^d^ Predictors: (Constant), years of service, race group, gender, job status, highest ed level, marital status and age group.

The results show that Model 4 (demographic variables—dedication and absorption combined) is not statistically significant at 5% levels of significance (*p*‐value = 0.598 > 0.05) indicating that less than 1.2% of the total variation in Model 4 is explained by the demographic variables. The impact of the demographic variables on Model 5 (demographic variables—absorption–dedication and vigour combined) is statistically significant at a 5% level of significance (*R* = 0.015 with a *p*‐value of 0.044 < 0.05) implying that 1.5% of the total variation in Model 5 is explained by demographic variables. Model 6 (demographic variables—absorption–vigour) is statistically significant at a 5% level of significance (*p*‐value = 0.002 < 0.01) indicating that 3.1% of the total variation in Model 6 is explained by demographic variables. As Models 5 and 6 were significant, further investigations were conducted (Table 5).

**TABLE 5:** Model fit indices for demographic variables on sub‐factors.

	Model 5 (Dependent variable: absorption—dedication and vigour combined)	Unstandardized B	Coefficients std. error	Standardized coefficients beta	*T*	Sig.
1	(Constant)	4.302	0.214		20.105	0.000
	Race group	0.009	0.020	0.020	0.441	0.659
	Age group	0.070	0.028	0.132	2.485	**0.013**
	Gender	−0.010	0.049	−0.009	−0.206	0.837
	Marital status	−0.071	0.034	−0.104	−2.118	**0.035**
	Highest ed. level	−0.087	0.055	−0.077	−1.595	0.111
	Job status	–0.046	0.178	–0.012	–0.256	0.798
	Years of service	–0.023	0.038	–0.037	–0.608	0.544

**Model 6 (Abs–Vig combined** a. Dependent variable: WE absorption–vigour comb						
1	(Constant)	4.439	0.303		14.630	0.000
	Race group	–0.043	0.029	–0.069	–1.494	0.136
	Age group	0.132	0.040	0.174	3.297	**0.001**
	Gender	0.065	0.070	0.043	0.939	0.348
	Marital status	–0.043	0.048	–0.044	–0.902	0.368
	Highest ed. level	–0.018	0.077	–0.011	–0.230	0.818
	Job status	–0.662	0.253	–0.121	–2.622	**0.009**
	Years of service	–0.092	0.053	–0.104	–1.735	0.083

*Note*: The bold numbers show the most significant effects.

Age holds the strongest effect on work engagement sub‐factors, as indicated in both Models 5 and 6. Model 5 presented age and marital status as having the strongest effect on absorption–dedication and vigour. Model 6 in turn identified age and job status as having a significant effect on absorption–vigour combined, implying that the older the employee, the higher the effect on absorption–vigour combined.

As depicted in Table 6, the fit indices meet the required thresholds as indicated by ‘acceptable’ and ‘good’ in the threshold column [43].

**TABLE 6:** Presentation of model fit indices.

Fit indicator	Threshold*	IMM	FMM
CMIN/DF (Chi‐square/degree of freedom)	Acceptable	6.998	3.341
RMSEA (RMSEA)	Acceptable	0.109	0.068
NFI	Acceptable	0.557	0.902
CFI	Good	0.593	0.929
TLI	Good	0.572	0.918
GFI	Acceptable	0.458	0.852
AGFI	Good	0.412	0.819

Abbreviations: AGFI, Adjusted goodness‐of‐fit‐index; CFI, Comparative fit index; GFI, Goodness‐of‐fit‐index; IMM, Initial measurement model; MM, Final measurement model; NFI, Normed fit index; RMSEA, Root mean square error of approximation; TLI, Tucker Lewis index

* Indicates the degree of model fit.

## DISCUSSION

Talent management holds much potential to support resource‐scarce healthcare settings [1, 11], but it is not used optimally by hospital management [8]. Our results identified significant differences between hospitals’ moderate levels of management commitment, talent review, workforce planning and staffing, versus the high‐importance nurses, attached to these practices. The perceived lack of commitment to developing talent is likely to cause nurses to withdraw and become disengaged, in response. The results also confirmed that acquiring, reviewing and planning for appropriate staffing levels are very important to support nurses’ engagement. Social exchange theory [27] explains that there is a reciprocal process at work, and if nurses’ expectations of appropriate staffing and development opportunities are not met, they will respond with decreased work engagement, as reflected in these results.

Our results provide ample evidence to support the framework presented (Figure 1).

All the talent management practices current‐status had a positive effect, with talent review, workforce planning and talent acquisition having a specifically noteworthy effect on dedication and absorption combined (Model 1). Planning and investing resources to ensure manageable workloads and nurse‐patient care ratios are therefore essential if nurses are to reciprocate and devote their cognitive, emotional and physical resources [21]

In addition, although less than for Models 1, 2 and 3 showed support for our claim that all the talent management practices importance‐rating have a positive effect on work engagement, in‐line with [44] findings. The results show that talent development and commitment combined has a strong effect on dedication and absorption, meaning that learning interventions, such as coaching and mentoring are likely to improve engagement [40]. This finding reflects positive psychology's emphasis on employees’ need for ‘growth’, and continuous learning and development opportunities, in this sample of nurses [32]

Lastly, we investigated the effect of ethnic group, age, gender, marital status, educational level, job status and years of service talent management status‐rating and work engagement (Models 4, 5 and 6). Our findings revealed nurses’ age and marital status have a significant effect on absorption–dedication and vigour combined, as shown by Model 5. Model 6 revealed that age and job status have a significant effect on absorption–vigour combined. These results emphasize the importance of providing a diverse range of development opportunities that are appropriate and attractive to nurses of all ages, marital and job statuses, to encourage their work engagement [22]

### Practical implications and recommendations

The results showed that not only does talent management improve work engagement, but also that nurses need to see that hospital managers are committed to investing resources in planning to achieve adequate staffing levels, continuous reviewing and developing talent. When managers visibly demonstrate their commitment to talent management, nurses reciprocate with continued high engagement and quality patient care. Following an inclusive and diverse talent development approach, management should offer bursary schemes to attract and ensure a more reliable supply of new, younger nurses, while also offering challenging job assignments, coaching, mentoring and management development programmes to established professionals [45]. Hospital managers should also consider that married nurses and caregivers may experience more work‐life balance conflicts, find it difficult to attend external learning events and prefer in‐house opportunities to prepare for future strategically important positions.

This study has some limitations. First, the study design was cross‐sectional and these are not robust predictors of causality; although the study contributes to our understanding of the effect of talent management within the healthcare sector. Second, the data was collected from three large public hospitals in Gauteng, South Africa, limiting the generalisability of the data.

## CONCLUSION

The findings of this study contribute to ongoing research on how strategic talent management practices can serve to improve work engagement in the healthcare sector. Attracting and retaining talented nurses are a global challenge, and this study offers novel insights, by integrating social exchange theory and positive psychology theory with talent management and work engagement findings. Hospital managers can improve the engagement of nurses by demonstrating their commitment to talent development, offering a diverse variety of development opportunities and ensuring a pipeline of new nurses by improving the attractiveness of the profession. Effective succession planning can also be used to ensure nurses remain energetic and dedicated to their patients. Future research following a longitudinal design, larger samples and other industries will provide helpful additional insights.

## AUTHOR CONTRIBUTION

*Conceptualization; data curation; formal analysis; investigation; methodology; project administration; writing—original draft; writing – review and editing*: Mpho Lerotholi. *Formal analysis; methodology; supervision; visualization; writing—review and editing*: Adele Bezuidenhout.

## CONFLICT OF INTEREST STATEMENT

The authors declare no conflict of interests.

## FUNDING INFORMATION

We would like to acknowledge and express our gratitude for financial support provided by the University of South Africa (Unisa) in the form of a postgraduate Bursary.

## ETHICS STATEMENT

Ethical approval was obtained and the certificate can be provided upon request.

## Data Availability

The data that supports the findings of this study are available in the supplementary material of this article.

## References

[1] Sopiah S , Kurniawan DT , Nora E , Narmaditya BS . Does talent management affect employee performance?: the moderating role of work engagement. ACS Food Sci Technol. 2020;7:335‐341.

[2] Bolander P , Werr A , Asplund K . The practice of talent management: a framework and typology. Emerald Publishing Limited; 2017.

[3] Hafez E , AbouelNeel R , Elsaid E . An exploratory study on how talent management affects employee retention and job satisfaction for personnel administration in Ain Shams University Egypt. J Manage Strategy. 2017;8:1.

[4] Rathnayake S , Dasanayake D , Maithreepala SD , Ekanayake R , Basnayake PL . Nurses’ perspectives of taking care of patients with Coronavirus disease 2019: a phenomenological study. PLoS One. 2021;16:e0257064. 34478482 10.1371/journal.pone.0257064 PMC8415609

[5] Dries N . Talent management, from phenomenon to theory. Hum Resour Manag Rev. 2013;23:267‐271.

[6] Vecchi A , Della Piana B , Feola R , Crudele C . Talent management processes and outcomes in a virtual organization. Bus Process Manag J. 2021;27(7):1937‐1965.

[7] Demerouti E , Bakker AB , Nachreiner F , Schaufeli WB . The job demands‐resources model of burnout. J Appl Psychol. 2001;86:499‐512. 11419809

[8] Saks AM . Antecedents and consequences of employee engagement. J Manag Psychol. 2006;21(7):600‐619.

[9] Seligman ME . The president's address. Am Psychol. 1999;54:559‐562.

[10] Knight C , Patterson M , Dawson J . Building work engagement: a systematic review and meta‐analysis investigating the effectiveness of work engagement interventions. J Organ Behav. 2017;38:792‐812. 28781428 10.1002/job.2167 PMC5516176

[11] Bakker AB , Albrecht S . Work engagement: current trends. Career Dev Int. 2018;23(1):4‐11.

[12] Al Aina R , Atan T . The impact of implementing talent management practices on sustainable organizational performance. Sustainability. 2020;12:8372.

[13] Hart KE , Sasso T . Mapping the contours of contemporary positive psychology. Can Psychol/Psychologie Canadienne. 2011;52:82‐92.

[14] Schaufeli W , Bakker AB . The conceptualization and measurement of work engagement. Psychology Press; 2010.

[15] Boamah SA , Read EA , Spence Laschinger HK . Factors influencing new graduate nurse burnout development, job satisfaction and patient care quality: a time‐lagged study. J Adv Nurs. 2017;73:1182‐1195. 27878844 10.1111/jan.13215

[16] Barkhuizen EN , Stanz KJ . Linking organisational energy and individual well‐being: The Influence of Leader's Talent Mindset. Conference Proceedings of the 12th Annual Conference of the Global Business and Technology Association, Kruger National Park, South Africa, 5–9 July, 2010. 50‐57. ISBN 1‐932917‐06‐3.

[17] Sheldon KM , King L . Introduction: why positive psychology is necessary. Am Psychol Assoc. 2001;56:216. 11315247

[18] Diedericks JC , Cilliers F , Bezuidenhout A . Resistance to change, work engagement and psychological capital of academics in an open distance learning work environment. SA J Hum Resour Manag. 2019;17:1‐14.

[19] Blake H , Bermingham F , Johnson G , Tabner A . Mitigating the psychological impact of COVID‐19 on healthcare workers: a digital learning package. Int J Environ Res Public Health. 2020;17:2997. 32357424 10.3390/ijerph17092997 PMC7246821

[20] Dzimbiri GL , Molefi A . The impact of talent management on job satisfaction of registered nurses in Malawian public hospitals. SA J Hum Resour Manag. 2021;19:9.

[21] Meyers MC , van Woerkom M , Paauwe J , Dries N . HR managers’ talent philosophies: prevalence and relationships with perceived talent management practices. Int J Hum Resour Manag. 2020;31:562‐588.

[22] Fahmi TM , Mohamed HAS . Examining the relationship between talent management practices, work engagement and intention to quit of academic staff: insights from Egyptian faculties of tourism and hotels. Int J Hosp Tour Syst. 2020;13:1‐12.

[23] De Boeck G , Meyers MC , Dries N . Employee reactions to talent management: assumptions versus evidence. J Organ Behav. 2018;39:199‐213.

[24] Malhotra N , Nunan D , Birks D . Marketing research: an applied approach. Pearson; 2017.

[25] Findlay P , Lindsay C , McQuarrie J , Bennie M , Corcoran ED , Van Der Meer R . Employer choice and job quality: workplace innovation, work redesign, and employee perceptions of job quality in a complex health‐care setting. Work Occup. 2017;44:113‐136.

[26] Yang K , Zhou L , Wang Z , Lin C , Luo Z . Humble leadership and innovative behaviour among Chinese nurses: the mediating role of work engagement. J Nurs Manag. 2019;27:1801‐1808. 31556172 10.1111/jonm.12879

[27] Field A . Discovering statistics using IBM SPSS statistics. sage; 2013.

[28] Organization WH . Delivering quality health services: a global imperative. OECD Publishing; 2018. 10.1136/ejhpharm-2018-001692 PMC6452396 31157041

[29] Mahfoozi A , Salajegheh S , Ghorbani M , Sheikhi A . Developing a talent management model using government evidence from a large‐sized city, Iran. Cogent Bus Manag. 2018;5:1449290.

[30] Cropanzano R , Mitchell MS . Social exchange theory: an interdisciplinary review. J Manage. 2005;31:874‐900.

[31] Barkhuizen N , Rothmann S , Van De Vijver FJ . Burnout and work engagement of academics in higher education institutions: effects of dispositional optimism. Stress Health. 2014;30:322‐332. 23949954 10.1002/smi.2520

[32] Drennan VM , Ross F . Global nurse shortages: the facts, the impact and action for change. Br Med Bull. 2019;130:25‐37. 31086957 10.1093/bmb/ldz014

[33] Pineau Stam LM , Spence Laschinger HK , Regan S , Wong CA . The influence of personal and workplace resources on new graduate nurses’ job satisfaction. J Nurs Manag. 2015;23:190‐199. 23844875 10.1111/jonm.12113

[34] Gabel‐Shemueli R , Westman M , Chen S , Bahamonde D . Does cultural intelligence increase work engagement? The role of idiocentrism‐allocentrism and organizational culture in MNCs. Cross Cult Strateg Manag. 2019;26:46‐66.

[35] Barkhuizen N , Stanz K , Van Rensburg NJ . The safe mindset of managers, shift bosses and miners on a South African platinum mine in South Africa. University of Johannesburg. 2010:508.

[36] Krishnan TN , Scullion H . Talent management and dynamic view of talent in small and medium enterprises. Hum Resour Manag Rev. 2017;27:431‐441.

[37] Castro‐Sánchez E , Santillán‐García A . Smart lobbying for minimum nurse staffing ratios in Spain: not just numbers. Policy Polit Nurs Pract. 2020;21:60‐61. 32370609 10.1177/1527154420923753 PMC7485012

[38] Ancarani A , Arcidiacono F , Mauro CD , Giammanco MD . Promoting work engagement in public administrations: the role of middle managers’ leadership. Public Manag Rev. 2021;23:1234‐1263.

[39] Lee YS , Nembhard IM , Cleary PD . Dissatisfied creators: generating creative ideas amid negative emotion in health care. Work Occup. 2020;47:200‐227.

[40] Bakker AB , Demerouti E . Job demands–resources theory: taking stock and looking forward. J Occup Health Psychol. 2017;22:273. 27732008 10.1037/ocp0000056

[41] McDonnell A , Collings DG , Mellahi K , Schuler R . Talent management: a systematic review and future prospects. Eur J Int Manag. 2017;11:86‐128.

[42] Kravariti F , Johnston K . Talent management: a critical literature review and research agenda for public sector human resource management. Public Adm Rev. 2020;22:75‐95.

[43] Lerotholi M . Talent Management, Work Engagement and Retention of professional nurses in Gauteng Academic Hospitals. PhD thesis, University of South Africa. 2021.

[44] Bagozzi RP , Yi Y . On the evaluation of structural equation models. J Acad Mark Sci. 1988;16:74‐94.

[45] Kahn WA . Psychological conditions of personal engagement and disengagement at work. Acad Manag J. 1990;33:692‐724.

